# Complex Care Needs in Multiple Chronic Conditions: Population Prevalence and Characterization in Primary Care. A Study Protocol

**DOI:** 10.5334/ijic.3292

**Published:** 2018-05-25

**Authors:** Francisco Hernansanz Iglesias, Clara Alavedra Celada, Carmen Berbel Navarro, Lidia Palau Morales, Nuria Albi Visus, Cristina Cobo Valverde, Vanessa Matias Dorado, Maria Luisa Martínez Muñoz, Carles Blay Pueyo, Esther Limón Ramírez, Raimon Milà Villaroel, Núria Montellà Jordana, Josep Maria Bonet Simó, on behalf of the SABCOMP group

**Affiliations:** 1 Catalan Institute of Health, Barcelona, ES; 2 Chronicity Prevention and Care Program, Department of Health, Government of Catalonia, ES; 3 University of Vic, Vic, Barcelona, ES; 4 The Qualy Observatory/World Health Organization Collaborating Centre for Palliative Care Public Health Programs, Catalan Institute of Oncology, L’Hospitalet de Llobregat, Barcelona, ES; 5 Pompeu Fabra University (UPF), ES

**Keywords:** primary care, integrated care, chronic diseases, patient complexity, complex care needs, multimorbidity

## Abstract

**Background:**

Chronicity, and particularly complex care needs for people with chronic diseases is one of the main challenges of health systems.

**Objective:**

To determine the population prevalence of people with chronic diseases and complex care needs and to characterize these needs considering features of health and social complexity in Primary Care.

**Design:**

Cross-sectional population-based study.

**Scope:**

Patients who have one or more chronic health conditions from three Primary Care urban centres of a reference population of 43.647 inhabitants older than 14 years old.

**Methodology:**

Data will be obtained from the review of electronical medical records. Complexity will be defined by: 1) the independent clinical judgment of primary care physicians and nurses and 2) the aid of three complexity domains (clinical and social). Patients with advanced chronic disease and limited life prognosis will be also described.

**Conclusions:**

This research protocol intends to describe and analyse complex care needs from a primary care professional perspective in order to improve knowledge of complexity beyond multimorbidity and previous consumption of health resources. Knowing about health and social complexity with a more robust empirical basis could help for a better integration of social and health policies and a more proactive and differentiated care approach in this most vulnerable population.

## Introduction

### Background/rationale

Aging and prevalence of multiple diseases as age increases is currently the norm rather than the exception and challenges the single disease model that dominates medical education, research and hospital care [1]. This phenomenon has given rise to terms such as comorbidity and multimorbidity, another challenge to primary care [2]. As programs or guidelines for chronic disease management focus on specific and single conditions, there is a growing concern that these programs may be less effective for individuals with multimorbidity compared to person-centered approaches [3]. In recent years a new concept, increasingly common in primary care, has been introduced: the “complex chronic patient” [4,5], which is defined by a particular profile presentation of chronicity where socio-economic, cultural and environmental dimensions play an essential role [6], reflecting person-specific factors interfering with the delivery of usual care and decision making and the need to implement specific individual plans [7].

Literature review about the complexity construct [8,9,10,11,12,13] has one feature in common: the presence of other dimensions, in addition to multimorbidity, that incorporate socioeconomic determinants of health, culture, environment, patient behaviour and their experience in the use of health services that evidence social inequalities in health [1]. Grant et al. suggest different complexity patterns where mental health and substance abuse were identified as the main problems in younger complex patients, while decision-making and care coordination predominated in advanced age [14]. Loeb et al. perceive complexity if patients have an exacerbating factor—a medical illness, mental disease, socioeconomic challenge, or behaviour or trait (or some combination thereof)—that complicate care for chronic health conditions [15].

Estimated prevalence of complexity ranges from 5% [16,17] to 24% [14]. Physician defined complexity has modest agreement with traditional comorbidity algorithms [14,18] meaning that patient complexity is probably a multifaceted concept that is not always adequately contemplated by the current multimorbidity groupers [17].

Within this context, the Chronicity Prevention and Care Programme (PPAC) (Health Plan for Catalonia 2011–2015 [19]) addresses two groups of complex patients: complex chronic patients (PCC) and patients with advanced chronic disease and life limited prognosis (MACA) [20]. Allocation of patients in each group depends on the clinical judgment of referent primary care professionals, considering clinical, psychosocial and stratification strategies or risk groupers, but there is no gold standard for unequivocal identification so far.

### Objectives

The aims of this protocol are:

1. To determine the population prevalence of people with chronic diseases and complex care needs (PCC and MACA), according to the construct proposed by Chronicity Prevention and Care Programme.
2. To characterize complex care needs, considering elements of health and social complexity, of people with chronic diseases in Primary Care.
3. To describe the frequency and distribution of Chronicity Prevention and Care Programme criteria to identify people with chronic disease and complex care needs.
4. To identify which Chronicity Prevention and Care Programme criteria would predict complexity better.
5. To relate complexity identification to professionals’ characteristics.

## Theory and Methods

### Study protocol design

Cross-sectional, population-based observational study and prospective cohort study for survival analysis.

### Study protocol population

The present study is carried out in the town of Sabadell, in the province of Barcelona (Spain), with an approximate population of 207,444 inhabitants [21], 48.6% male, with a percentage of those over 65 and under 14 years old of 18% and 16% respectively. The study focuses on three urban primary care centres of the unified Unit of Management of Primary Care (UGAP) Nord, managed by the Institut Català de la Salut (ICS) (Table 1). Participating practices have different socioeconomic backgrounds (lower, middle-lower, middle-upper and upper classes) according to the MEDEA deprivation index, validated in Spain and based on urban socioeconomic indicators in the Spanish census [22]. The bigger the Medea index, the worst the socioeconomic situation is. Medea index is calculated using five census-based socioeconomic indicators (percentages, by census tract): 1) unemployment rate, 2) manual workers, 3) temporary workers, 4) illiterate adults (or less than basic, mandated education), and 5) school drop-outs among the population less than 16 years old.

**Table 1:** Participating Primary Care practices distribution.

Primary Care Centre	POPULATION ≥14 years old	Attended	%>75 years old	MEDEA INDEX

NORD	13700	84.04%	9.22%	1,56
CA N’ORIAC	17198	82.17%	9.97%	1,41
CONCORDIA	12749	82.57%	9.37%	0,49
Total	43647			

### Study protocol subjects

The study analyses complexity in patients older than 14 years old, registered at the three practices, with a Clinical Risk Group (CRG) ≥ 5 that means they suffer from at least one chronic condition.

### Data collection

From the central information systems service, all the patients assigned to each health professional (physician and nurse) who meet the inclusion criteria will be gathered and an administrative assistant will create an agenda that will last for a three months recruitment period. During this period of time, physicians and nurses will review independently the electronic medical record and complete the case report form for each patient. Health professionals recently integrated (less than a year) into the primary care workforce, due to lack of patient list knowledge regarding social and health background, are excluded from the study. All professionals included decide to participate voluntarily and there is no remuneration for participating and collecting the study data.

### Chronic complex patient (PCC) and advanced chronic disease and life limited prognosis (MACA) definitions

Chronic complex patient will be defined by: 1) the independent clinical judgment of each assigned physician and nurse. The identification of one patient as complex may or may not match between physician and nurse, and 2) the aid of three domains of complexity designed by the Chronicity Prevention and Care Programme [23] (Table 3) and adapted to the study. Of those identified as complex there will be a group characterized as advanced chronic disease and life limited prognosis by the “surprise question”: Would you be surprised if this patient dies within one year?, contained in the NECPAL-CCOMS-ICO© instrument [24].

### Variables

The case report form contains demographic, clinical and social information. Tables 2 and 3 describe the questionnaires.

**Table 2:** Demographic and clinical data available in electronic health records.

IDENTIFICATION, patient ID		

Referent Physician		
Referent Nurse		
Clinical Risk Group	See description in text	
Risk of admission	See description in text	
Adjusted Morbidity Group	See description in text	
Age		
Previously identified as “Advanced chronic disease and life limited prognosis” (MACA)	Yes □	No □
Previously identified as “Complex Chronic Patient” (PCC)	Yes □	No □

**Table 3:** Chronicity Prevention and Care Programme (PPAC) criteria.

COMPLEXITY CRITERIA	ANSWER

**Patient-dependent criteria (PPAC)**	

Multimorbidity (≥2 chronic diseases)	Yes/No/Don’t know
A single severe chronic disease (including advanced frailty states)	Yes/No/Don’t know
A chronic progressive disease	Yes/No/Don’t know
High probability of undergoing decompensations with many symptoms and poor control	Yes/No/Don’t know
Patient with very dynamic evolution, variable, who needs continuous follow-up	Yes/No/Don’t know
High utilization of health services (emergency, Primary care appointments)	Yes/No/Don’t know
Polypharmacy (≥ 5 medicines) and/or high cost of resources	Yes/No/Don’t know
Frail patients with functional loss, probability of acute deterioration (functional or cognitive) or new onset of geriatric syndromes	Yes/No/Don’t know
**Professional Dependent criteria (PPAC)**	

Need for multidisciplinary hospital management	Yes/No/Don’t know
Need to activate and manage access to different resources (often by preferred routes)	Yes/No/Don’t know
Environment of special uncertainty in the decisions and doubts in clinical management	Yes/No/Don’t know
**Social complexity**	

Patient with adverse psychosocial situations (PPAC)	Yes/No/Don’t know
Patient whose management would benefit from integrated care strategies (PPAC)	Yes/No/Don’t know
Patient with relational problems	Yes/No/Don’t know
Patient with economic problems	Yes/No/Don’t know
Patient with loss of functional autonomy	Yes/No/Don’t know
**Other criteria**	

Patient with chronic neurological disease	Yes/No/Don’t know
Patient with severe mental disorder	Yes/No/Don’t know
Patient with dementia	Yes/No/Don’t know
Patient with psychic disability	Yes/No/Don’t know
Elderly patient (≥75 years old)	Yes/No/Don’t know

Clinical Risk Groups (CRG) is a population classification system that uses inpatient and ambulatory diagnosis and procedure codes, pharmaceutical data and functional health status to classify each individual into a hierarchically defined health status group. CRG classifies people into one of the following health states: 1) Healthy; 2) Significant acute illness; 3) Single or multiple minor chronic disease; 4) Moderate chronic diseases; 5) Chronic dominant diseases; 6–7) Multiple dominant chronic diseases; 8) Advanced neoplastic disease; 9) Catastrophic diseases. Its purpose, among others, is to detect diseases requiring greater attention, to monitor prevalence rates of chronic diseases, to understand the patterns of use and consumption of services and to develop risk and price adjustment applications [25].

The Adjusted Morbidity Groups (GMA) is a new multimorbidity risk adjustment grouper developed and adapted to the Spanish healthcare system and fully implemented into primary care clinicians’ workstation by May 2015. The GMA classifies individuals into unique and mutually exclusive groups taking into account: (i) type of disease, (ii) occurrence of multimorbidity; and, (iii) case complexity. Four pyramidal strata are identified and the higher the patient is in the pyramid, the more complexity, the more severity and the higher the risk of mortality and hospital admission. The GMA-1 or low-risk stratum corresponds to 50% of the population with a lower complexity level; GMA-2 or moderate risk stratum: 30% of the population, which has higher complexity than the previous risk stratum.; GMA-3 or high-risk stratum: corresponds to 15% of the population, which is above GMA-2 stratum, GMA-4 or very high risk stratum: 5% of the population, which has the highest complexity level [26]. GMA has been recently adopted by 13 of the 17 regional healthcare systems in Spain, covering 92% of the overall Spanish population, approximately 38 million citizens.

Risk of admission: Probability of urgent admission in the following twelve months adjusted for age, sex, socioeconomic status (MEDEA) and morbidity (GMA). The methodology applied is a logistic regression (with urgent or not urgent admission) and depending on the probability obtained, different risk levels are assigned.

### Statistic analysis

A descriptive statistical analysis will be carried out. Characteristics of complex versus non-complex patients will be compared using chi-square or t-test as appropriate. The characteristics of professionals (years in practice at the centre, years since specialty, age, sex, teaching, size of patient panel etc.) will be included. The agreement between physicians and nurses when defining complexity will be measured using the Kappa statistic. A multivariate logistic regression model will be constructed to identify independent predictors of complexity by adjusting for different variables. Multiple correspondence analysis will be used to identify the clustering patterns of complexity. Complex chronic patients and patients with advanced chronic disease and life limited prognosis survival times (times-to-event) will be estimated with Kaplan-Meier curves.

### Confidentiality and ethical approval

The study protocol will be carried out in accordance with current legal regulations. This project has been approved by the Clinical Research Ethics Committee (CEIC) of reference, Institute of Research in Primary Care (IDIAP) Jordi Gol i Gorina (Barcelona) with reference number P15/119.

As there are no intervention on patients nor additional information required, only the one in the shared electronic health records to fill in the case report form performed by its reference professionals, it is not considered necessary for the participating patients to give their informed consent.

The study investigators are committed to comply with the Organic Law of Data Protection. As the database will be anonymized, none of the data collected could be used to identify patients.

## Discussion

### Strengths

The conceptual model of complexity has not yet been satisfactorily translated into medical practice. The main strength of this research is the involvement of Primary Care physicians and nurses in defining complex (social and health care) needs beyond multimorbidity and current risk adjustment. The existence of list of patients registered with a professional and its longitudinality allow to understand patients’ complex milieu of medical, mental health and social issues, helps to prioritize these issues and assist patients to make healthcare decisions in alignment with their goals [27]. Multimorbidity is now the norm and primary care professionals cope with an average of more than three health problems at each health encounter [28]. The complexity of outpatient consultations, understood by the number of diagnoses reported, is usually higher for primary care physicians than for other medical specialities [29]. The literature on care co-ordination for older people with complex medical problems and/or multimorbidity places high importance on the role of primary care (multidisciplinary team work, medical home) [30]. Spain has strong primary care services [31] and countries with such primary care (patient-centred care, access, longitudinality and care coordination with other providers) tend to perform better in chronic care management [32].

Unmet social needs have an impact on health, longevity and health spending, and reveal the need to redesign health systems and implement proactive strategies for social and healthcare integration [33]. It is urgent to move from a fragmented framework, where the individual receives and requests benefits to social and health services separately, to a new model of shared responsibility and collaborative practices.

The availability of better population risk-adjustment tools and deeper knowledge in complex care needs will make possible to anticipate in advance those patients who are going to benefit the most from proactive health and social care. To date, only one study has defined the complexity of the patient from the perspective of a primary care professional [14]. Another study has added qualitative detail that supports and expands on the previous cohort study by Grant et al [15].

Our proactive identification is clearly aligned with the strategy of prevention and care for complex chronic patients defined in the Catalan Health Plan for the next years and can offer external validity within our National Health System.

### Limitations

The use of electronic health records could be limited by missing, incorrect or unverified data. The prevalence of multimorbidity is influenced by the quality of electronic health data or the introduction of risk factors (obesity, lipid disorders) as diseases. As patients included in the research must be in the Clinical Risk Group heath state ≥ 5, patients with single or multiple minor chronic diseases are not taken into account.

Primary care’s perspective on complexity may be influenced by other providers (different hospital admission criteria not related to morbidity and admission rates explained by bed supply-availability rather than by any care need variable), available resources within the health area, vocational training, professionalism, and coordination with other health workforces such as specialists and social services [34].

In the absence of a gold standard to describe patient complexity, the results should be understood from the perspective of Primary Care workforces. And how social and economic background knowledge can influence health complexity. We reflect the perspective of Primary Care professionals while other research has developed a definition of complexity focused on patients’ functional status: the balance between patient workload of demands and patient capacity to address demands [12].

The inter-observer variability has been limited by training actions carried out with the case report form. The results may not be extrapolated to other Primary Care scenarios, especially where longitudinality of patients is not favoured.

In this study the paediatric population younger than 14 years old is excluded. No rural practices were included.

## Conclusions

This protocol aims to describe and analyse the complex care needs from the perspective of the primary care professional and to improve the knowledge of complexity beyond multimorbidity and prior consumption of health resources. Defining health and social complexity could help for a better integration of social and health policies, lay the groundwork basis of future Health Plans, and a more proactive and differentiated care approach in this most vulnerable population.

## Previous presentations

The approach used in this project was presented at the Catalan Health Plan Congress 2015–2020 and at the International Conference on Integrated Care (ICIC16) held in May 2016 in Barcelona, Spain.
